# Environmentally sensitive fluorescence of the topical retinoid adapalene

**DOI:** 10.3389/fchem.2024.1438751

**Published:** 2024-07-08

**Authors:** Juan A. Soler-Orenes, Antonio Monari, Miguel A. Miranda, Javier Hernández-Gil, Virginie Lhiaubet-Vallet

**Affiliations:** ^1^ Instituto Universitario Mixto de Tecnología Química (UPV-CSIC), Universitat Politècnica de València, Consejo Superior de Investigaciones Científicas, Valencia, Spain; ^2^ Université Paris Cité and CNRS, ITODYS, Paris, France

**Keywords:** albumin, DNA, micelles, micropolarity, steady-state fluorescence, topical drug

## Abstract

Intrinsic fluorescence of drugs brings valuable information on their localization in the organism and their interaction with key biomolecules. In this work, we investigate the absorption and emission properties of the topical retinoid adapalene in different solvents and biological media. While the UVA/UVB absorption band does not exhibit any significant solvent-dependent behavior, a strong positive solvatochromism is observed for the emission. These results are in line with molecular modeling and simulations that show the presence of two quasi-degenerate states, i.e., a local π-π* and an intermolecular charge-transfer (ICT) state. However, molecular modeling also revealed that, whatever the solvent, at the corresponding equilibrium geometry the lowest and emissive excited state is the local π-π*. Finally, the potential of adapalene to act as a biological probe is demonstrated using albumin, DNA and micelles.

## 1 Introduction

The understanding of micropolarity in biological systems is critical to unveil processes such as protein folding, enzymatic functions, or in a more general way the ligand-biomolecule interactions. Fluorescent probes provide an easy and universal toolbox and their structure has been optimized over the years to offer a broad range of applications (Qin et al., 2021; Tian et al., 2021; Georgiev et al., 2023; Klymchenko, 2023). In this context, drugs containing a fluorophore present an added value as their intrinsic emission can bring direct and valuable information on their localization in biological media, and their affinity with proteins, membranes, or DNA (Nuin et al., 2011; Lammers et al., 2012; Lammers et al., 2013; Phetsang et al., 2016; Kabir et al., 2022).

**FIGURE 1:**
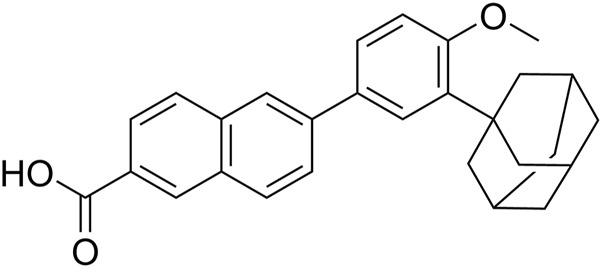
Adapalene chemical structure.

Adapalene, namely 6 [3-(1-adamantyl)-4-methoxyphenyl]-2-naphthoic acid (Figure 1), is a synthetic, third-generation topical retinoid derived from naphthoic acid (Rusu et al., 2020). It is commercialized as a topical formulation containing 0.1% or 0.3% drug in gel or cream for the treatment of acne vulgaris (Thielitz et al., 2008). Besides, adapalene effectivity has also been demonstrated in the therapy of epidermal proliferative diseases and proposed as a candidate for the clinical treatment of melanoma, prostate, or colorectal cancer. This activity is mainly associated with its role as a specific modulator of retinoic acid receptors (RARs) or retinoid X receptors (RXRs) that regulate cell growth, differentiation, survival, and death (Ocker et al., 2003; Shi et al., 2015; Li et al., 2019; Rusu et al., 2020; Wang et al., 2020; Nong et al., 2022).

Adapalene displays two significant structural features. They are related to the presence i) of the adamantyl group, an important pharmacophore existing in a large variety of pharmacologically active compounds (Liu et al., 2011), and ii) of the naphthalene moiety that confers it intrinsic fluorescence (Dai et al., 2023; Irshad et al., 2023). Yet, by a closer look at its structure, it appears that the aromatic core is substituted at position 2 with an electron withdrawing carboxylic group, and at position 6 with *p*-methoxyphenyl as an electron donating group. A parallel can be drawn with the structure of Prodan, i.e., 2-(dimethylamino)-6-propionylnaphthalene, which is among the most used environmental sensitive fluorescent probe (Qin et al., 2021). This naphthalene-derived push-pull dye owes its solvatofluorochromic properties to the presence of electron-donating and electron-withdrawing groups connected by the aromatic π-system. After excitation, an intramolecular charge-transfer (ICT) occurs as the electron density shifts from the donor to the acceptor through the π-conjugated linker. As a result, the dipole moment in the excited singlet manifold is increased with respect to that of the ground state, making the energy gap between these two states largely dependent to the environment polarity. Therefore, such solvatochromic dyes can not only sense polarity changes between different solvents or solvent mixtures but also the local variation of polarity in proteins, DNA, lipid membranes, or cellular organelles, which are often associated with important biological events (Zhu et al., 2016; Tian et al., 2021; Klymchenko, 2023).

Up to now the fluorescence of adapalene has been employed to investigate the drug stability under different stress conditions (Tolba and El-Gamal, 2016), or to evaluate its dermal distribution and release behavior (Allec et al., 1997; Brammann et al., 2020). Here, the environment-dependent properties of adapalene singlet excited state are addressed in different media by fluorescence spectroscopy and computational chemistry. Taking advantage of the pronounced emission shift obtained between polar and nonpolar solvents, the ability of adapalene to act as a fluorescent probe, to investigate its location in biological media, is also explored using human serum albumin, DNA, or micelles as biological hosts.

## 2 Materials and methods

### 2.1 Chemicals

Adapalene was purchased from BIOSYNTH Carbosynth and was used without further purification. All solvents (HPLC grade) were obtained from Scharlab. Human Serum Albumin (HSA), deoxyribonucleic acid (DNA) sodium salt from salmon testes, hexadecyltrimethylammonium bromide (CTAB) and phosphate buffered saline (PBS) tablets were purchased from Merck Life Science S.L.U.

### 2.2 UV-vis absorption spectroscopy

Absorbances of the samples were measured with a single beam Cary 60 Scan (Varian) UV-vis model spectrophotometer, using 1 cm pathway quartz cuvettes.

### 2.3 Fluorescence emission

Emission spectra of adapalene were obtained on a FLS1000 spectrometer (Edinburgh Instruments) equipped with a 400 W Xenon lamp, double grating Czerny-Turner monochromators with 2 mm × 325 mm focal length in excitation and detection, and a PMT-980 detector in a cooled housing which covers a range from 200–980 nm. The samples were prepared in different solvents, with an absorbance of 0.05–0.25 at the excitation wavelength, λ_exc_. Time-resolved fluorescence was also performed using the FLS 1000 system using EPLED 320 (λ_exc_ = 313 nm) as excitation source.

Experiments with biological components were performed in PBS at pH 7.4, using a final concentration of adapalene of 10 μM with 0.1% of DMSO (v/v) (from a 10 mM stock solution in DMSO) and an excitation wavelength of 315 nm. All the final samples were kept 30 min in the dark before performing the measurements. A stock solution of HSA (200 μM) was prepared in PBS. Then, different amounts of this solution were added to a cuvette containing 1.5 mL of 20 μM adapalene, and filled to 3 mL with PBS.

For DNA experiments, the concentration in base pair (bp) was obtained using the molar absorption coefficient at 260 nm, ε_260_ = 13,200 cm^−1^ M^−1^, concentrations from 25 to 400 μM in bp and 10 μM adapalene were used. Fluorescence quenching studies were run by gradually adding a KI solution to adapalene (10 μM) alone in PBS or in the presence of DNA (10 and 100 μM in bp). In the case of CTAB, buffered solutions containing 10 μM of adapalene and CTAB (from 1 to 10 mM) were prepared.

### 2.4 Molecular modeling

The ground state geometry of adapalene was optimized using density functional theory (DFT) approach in gas phase. The geometry optimization was performed using the Orca5.0 code (Neese, 2012; Neese et al., 2020) using the ωb97X-D functional including the Grimme corrections for dispersion (Chai and Head-Gordon, 2008) and the def2-TZVP basis set. Resolution of the identity in the RIJCOSX approach as implemented in Orca was consistently used. The geometry optimization was repeated in different solvents, which were modeled with the polarizable continuum approach (Mennucci, 2012), namely hexane, THF, dichloromethane, ethanol, DMF, and acetonitrile. Absorption spectra were calculated on top of the ground state equilibrium geometries using the time-dependent DFT (TD-DFT) approach in the Tamm Dancoff approximation. The same level of theory as for the ground state optimization was used, and five vertical transitions were converged. The vertical transitions were convoluted using Gaussian functions of fixed width at half-length of 0.3 eV to better reproduce experimental spectra. The diabatic nature of the excited states was determined by calculating natural transition orbitals (NTOs) (Martin, 2003). The geometry of the two lowest excited states was optimized once again using the same level of theory and the equilibrium PCM approach to simulate emission spectrum. As concern the modeling of the solvent environment we have chosen to consider the simplest system, i.e., isolated molecule embedded in a polarizable continuum. This choice is dictated by different factors: firstly, since the molecules maintain its rather rigid arrangement we believe that the interaction with biological media should not significantly alter its geometrical parameters. On the other hand we have excluded explicit microsolvation since we are mostly interested by non-protic solvent and in this extent PCM methods may already reproduce correctly the behavior of the different excited states in response to the different solvent polarity, even if a larger shift between experimental and simulated absorption and emission maxima may be observed.

## 3 Results and discussion

### 3.1 UV-vis absorption spectrometry

**FIGURE 2:**
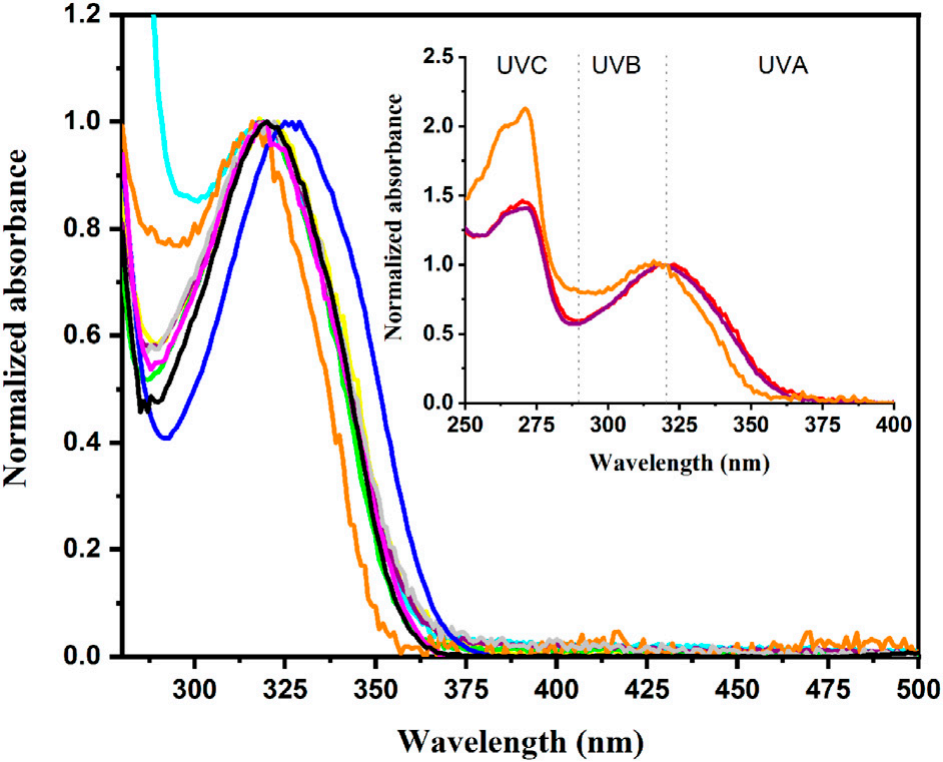
Normalized UVA absorption band in the different solvents. (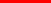 acetonitrile, 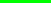
ethyl acetate, 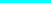
1,4-dioxane, 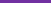
ethanol, 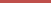
hexane, 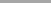
methanol, 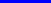
dichloromethane, 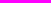
DMF, 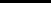
THF). Inset: Normalized UV-vis absorption spectra of adapalene in acetonitrile, ethanol and hexane.

As a first step, the absorption spectra of adapalene were registered in different solvents. They exhibit two absorption maxima, one is centered in the UVC region whereas the second one peaks at the limit between UVB and UVA (see Figure 2 inset for acetonitrile, ethanol and hexane). The absorption spectrum was found to be relatively insensitive to the solvent polarity, with only a small red shift of the long wavelength absorption band from 317 nm in hexane to 329 nm in dichloromethane (Table 1; Figure 2). The presence of this low energy absorption band is meaningful for a topical drug because UVA is considered as the photobiologically active region and is involved in many photosensitization processes leading to detrimental effects on living organisms (Improta and Douki, 2021; Baptista et al., 2023). As a matter of fact, UVA absorption allows the selective excitation of adapalene in complex biological matrices, and specifically with the simultaneous presence of biomolecules such as DNA or proteins, which is also a key requirement for acting as a biological relevant probe.

**TABLE 1:** Absorption (λ_A_, nm) and fluorescence (λ_F_, nm) maxima, Stokes shift (Δν, cm^−1^) of adapalene in solvents of different polarity (dielectric constant, є, refractive index, n, and E_T_ (30) parameter).

Solvent	є	*n*	E_T_ (30)	λ_A_	λ_F_	Δν/10^3^
Hexane	1.89	1.380	31	317	374	4.81
1.4-Dioxane	2.21	1.422	36	322	387	5.22
Ethyl acetate	6.02	1.370	38.1	319	396	6.10
THF	7.60	1.408	37.4	317	393	6.10
Dichloromethane	8.93	1.424	40.7	329	424	6.81
Ethanol	24.55	1.361	51.9	320	427	7.83
Methanol	32.65	1.329	55.4	322	441	8.38
DMF	36.70	1.428	43.2	324	420	7.05
Acetonitrile	37.50	1.344	45.6	320	429	7.94

**FIGURE 3:**
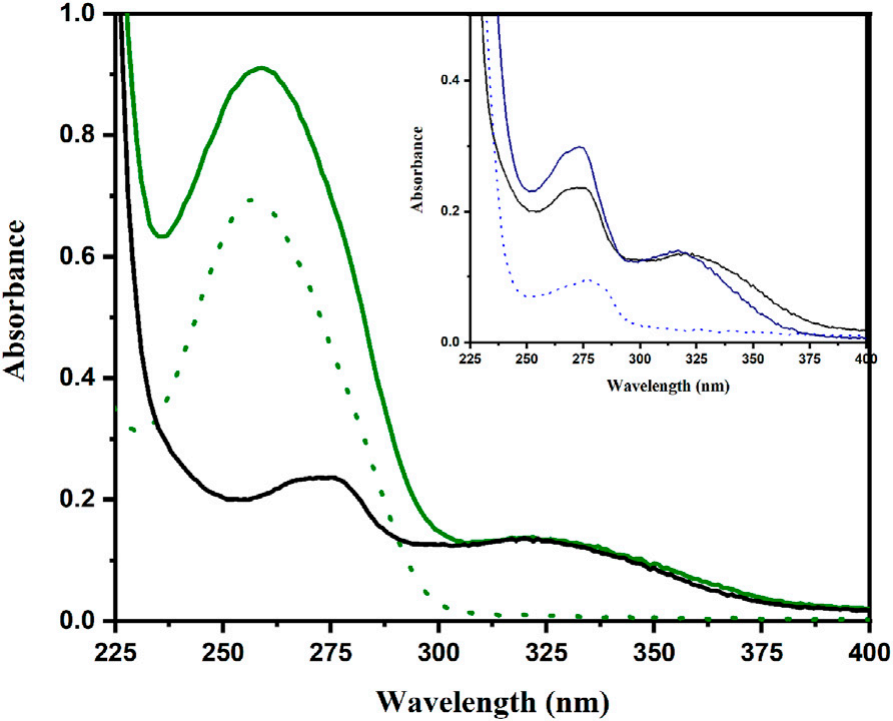
Absorption of adapalene alone (10 μM, gray), DNA (50 μM in bp, dotted green line) or adapalene in the presence of DNA (full green line) in PBS at pH 7.4. Inset: adapalene alone (10 μM, gray), HSA alone (2 μM, dotted blue line), of adapalene in the presence of HSA (full blue line).

Therefore, absorption spectrum of adapalene (10 μM) was also registered in the presence of human serum albumin (HSA), duplex DNA and CTAB micelles as a cellular membrane mimic. As observed in Figure 3, wavelengths from 300 to 370 nm are providing a valuable range for selective excitation of the drug avoiding direct irradiation of the biomolecule. No significant difference was observed in the case of DNA, whereas adapalene absorption experienced a blue shift in the presence of HSA (Figure 3 inset). This blue shift, as occurs for low polarity solvents such as hexane or THF (Table 1), is in line with its interaction with the less polar environment of the protein binding sites.

**FIGURE 4:**
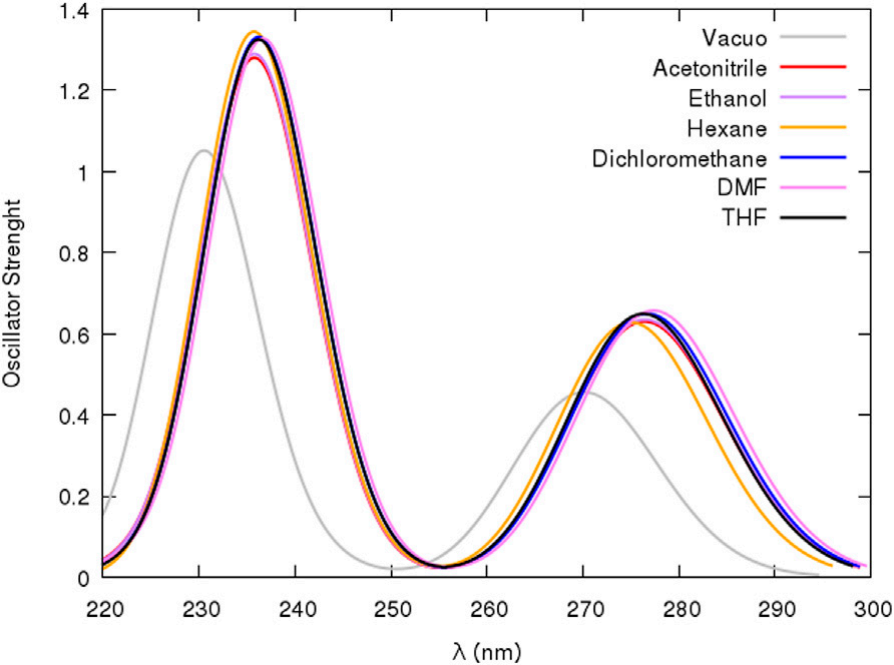
TD-DFT computed absorption spectrum in different solvents.

**FIGURE 5:**
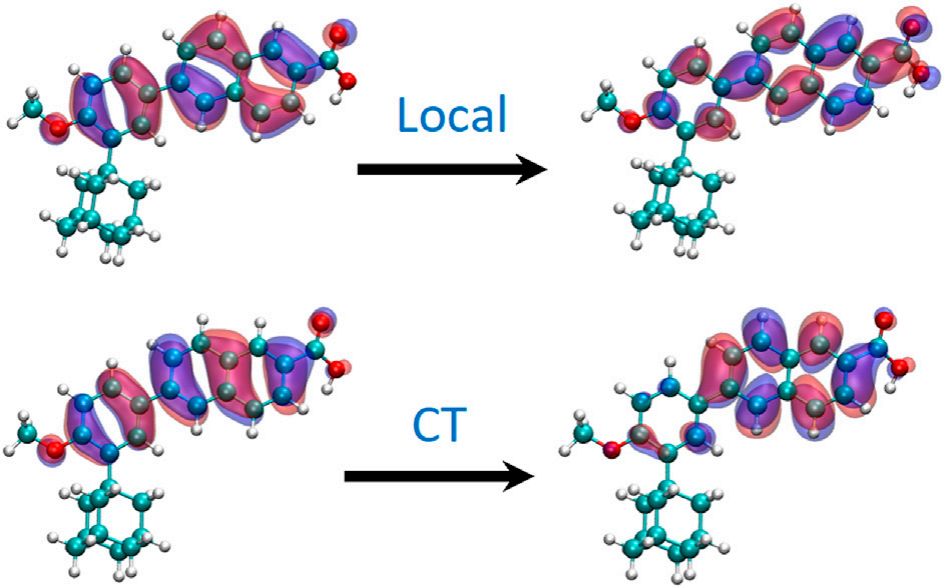
NTOs defining the two lowest excited states at Franck-Condon geometry.

These results are also globally confirmed by molecular modeling and simulations as shown in (Figure 4), which reports the absorption spectrum modeled as vertical transitions from the ground state equilibrium geometry, i.e., the Franck-Condon region. Notably the negligible role of the solvent in shifting the absorption maximum can also be noticed. Note that the shift compared to the experimental values can be considered acceptable for TD-DFT modeling performed statically from the equilibrium geometry only and neglecting vibronic couplings and flexibility. Interestingly the UVB/UVA band is due to two quasi-degenerate π-π* states having a profoundly different nature, i.e., locally excites or ICT, as can be seen from the NTOs reported in Figure 5. As can easily observed comparing the respective ocillator strenghts, At Franck-Condon and *in vacuo* the ICT state is the lowest, however the locally excited state is separated by less than 0.1 eV. As concern the ground state geometry, it is also important to notice that a significant deviation from planarity may be observed (Supplementary Figure S1) leading to a dihedral of about 138° between the phenyl and the naphthalene units. This geometric effect may also participate to favor the population of the ICT state due to the partial breaking of the extended π-conjugation.

### 3.2 Fluorescence emission

**FIGURE 6:**
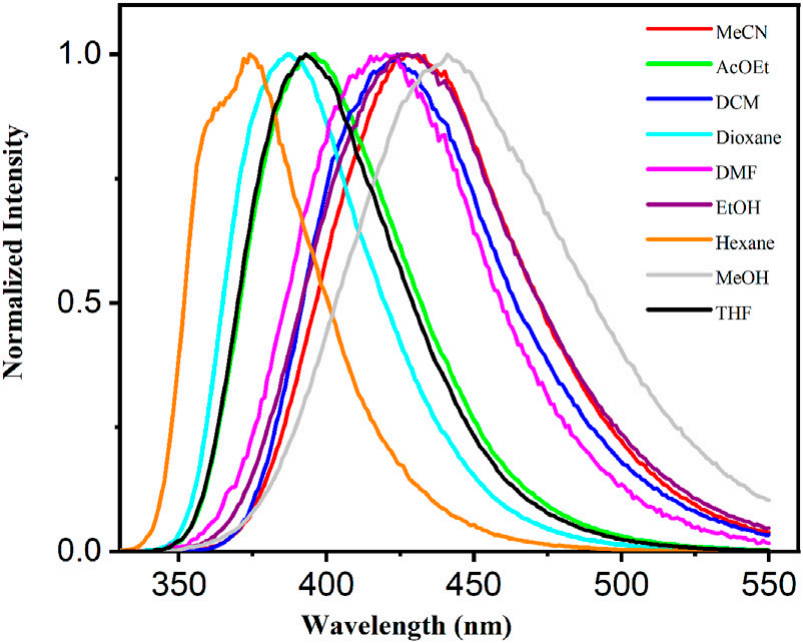
Normalized fluorescence spectra of adapalene in different solvents.

Singlet excited state characterization was further performed by steady-state and time-resolved fluorescence in different media. A solvent-dependent emission was evidenced (Figure 6) as the maximum suffers a bathochromic shift when solvent polarity increases moving from λ_F_ = 374 nm in apolar solvents such as hexane to 441 nm in methanol as polar media (Table 1; Figure 6). No clear tendency was observed in the emission lifetimes (τ_F_), values of τ_F_ of ca. 3.3, 2.3, and 4.1 ns were determined in hexane, acetonitrile and ethanol.

**FIGURE 7:**
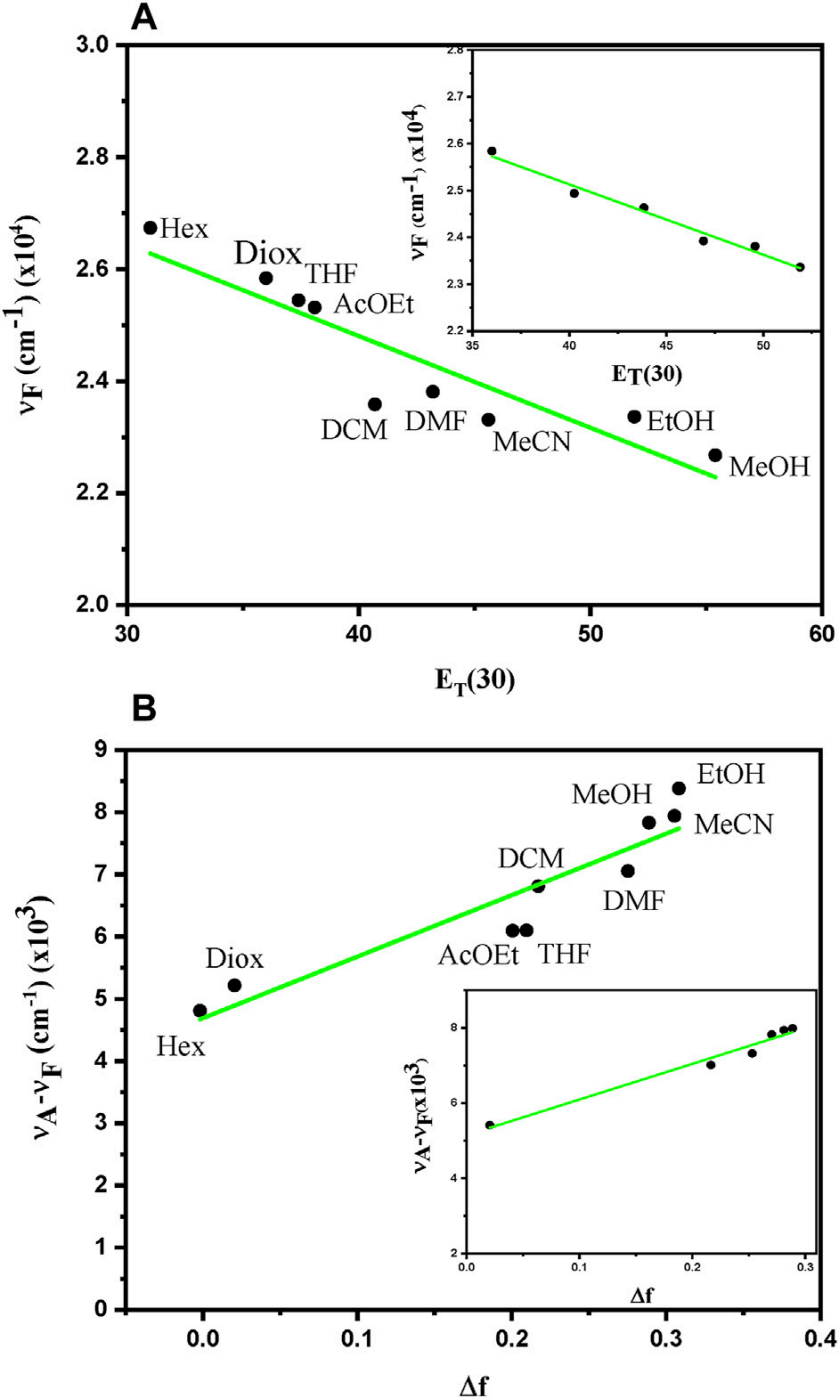
**(A)** Position of the emission maxima in wavenumber of adapalene *versus* the polarity E_T_ (30) in different solvents or in dioxane:ethanol mixtures (inset). **(B)** Lippert-Mataga plot for adapalene in different solvents or in dioxane:ethanol mixtures (inset).

A common method used to investigate the influence of the solvent polarity on emission properties of fluorophores consists in representing the band position (*ν*_F_ = 1/λ_F_ in cm^−1^) as a function of the polarity index ET (30) (Table 1). As shown in Figure 7A a linear correlation was found with a fitted slope of ca. 160 cm^−1^, no deviation is observed for protic solvents such as ethanol or methanol. Interestingly, this value is similar to that of the established naphthalene-derived probe PRODAN, which indicates the potential use of adapalene as an environment probe (Kucherak et al., 2010). Further experiments were performed using different percentages of dioxane-ethanol mixture, in this case the ET (30) mix values of the mixture were obtained from Eq. 1 where χ_Diox_ and χ_EtOH_ are the molar fraction of dioxane and ethanol in the mixture, ET (30)_Diox_ and ET (30)_EtOH_ are their polarity index given in Table 1.

$$E_{T}{\left(30\right)}_{mix}={\chi_{Diox}E}_{T}{\left(30\right)}_{Diox}+{\chi_{EtOH}E}_{T}{\left(30\right)}_{EtOH}$$ (1)

The linear dependence between *ν*_F_ and ET (30) was confirmed (Figure 7A, inset), and a similar slope of ca. 150 cm^−1^ was determined from the linear regression.

Another important characteristic describing polarity dependent fluorescence is based on the dipole interaction theory of Lippert and Mataga (Eq. 2), which predicts a linear correlation between the Stokes shift (Δμ) and the solvent polarizability (Δf) (Lippert, 1955). Indeed, solvatochromism may be related to the difference in the dipole moments (Δμ = μ_E_-μ_G_) of the fluorophore in the excited and ground states, the higher emission shifts corresponding to the larger Δμ. This value can be estimated from the Lippert-Mataga plot (Lippert, 1955; Mataga et al., 1956; Lakowicz, 2007), which represents the Stokes shift of the fluorescence emission (Δμ), expressed in wavenumbers, *versus* the solvent polarity (ϵ), and can be fitted using the Eq. 2.

$$\nu_{A}-\;\nu_{F}=\Delta \nu =\frac{2}{hca_{0}^{3}}\;\left(\frac{\epsilon -1}{2\epsilon +1}-\;\frac{n^{2}-1}{2n^{2}+1}\right)\;\left(\mu_{E}-\;\mu_{G}\right)=\frac{2\Delta f}{hca_{0}^{3}}\;\Delta \mu^{2}$$ (2)

Where ϵ and n are the solvent dielectric constant and refraction index, c is the velocity of light, h the Plank’s constant and a_0_ is the radius of the Onsager cavity. The term Δf encompassing ϵ and n dependence is known as the solvent polarizability. The Onsager cavity radius (a_0_) and ground state dipole moment (µ_G_) were calculated *in vacuo* by optimization of adapalene at DFT level and the values were estimated at 6 Å and 6.6 D, respectively. The Lippert-Mataga plot (Figure 7B) constructed from the data collected in different solvents revealed a linear relationship between Δ*ν* and the solvent polarizability Δ*f*, with a slope of ca. 9,900 cm^−1^. Again, no evidence was found for specific effect of protic solvents such as ethanol or methanol for which no significant deviation from the fitting was observed. Similar results were also obtained for dioxane-ethanol mixtures (Figure 7B, inset). Using the slope (Δ*ν*/Δ*f* = 9,900 cm^−1^) of the linear fitting and the calculated values of a_0_, a high Δμ = 14.6 D was determined from Eq. 2, showing an important increase between the dipole moments.

To rationalize these findings, we also optimized the two lowest excited states in different solvents to simulate the evolution of the emission spectroscopy. Importantly, whatever the solvent, the locally excited state was always the lowest and, thus, the emission bright state. Globally the energy gap between the lowest excited states at the S_1_ equilibrium geometry is between 0.4 eV for apolar media and 0.6 eV for polar solvents, while at Franck-Condon the two states are separated by 10^−3^–10^−2^ eV. Importantly, no evidence of state mixing may be observed and the local nature of the emissive state can be strongly confirmed. This is also coherent with the globally continuous evolution of the emission with the polarity of the solvent. The bathochromic shift due to the solvent and the increased Stokes shift are also confirmed by the computed values reported in Supplementary Table S1, even if some global shift of the simulated values may be observed. This can again be ascribed to the limited model, which neglects the dynamic and vibrational relaxation, and to the description of the solvent by a continuum model. However, our results confirm and corroborate the interpretation of the experimental data. In addition, the higher oscillation strength observed for the lowest state is also coherent with both its local character and the relatively high fluorescence quantum yield. Yet some questions may arise concerning the interpretation of the Lippert-Malaga plot. Indeed, the high difference of the dipole moment between the ground and excited states would suggest the presence of a charge-separated state, which is however contradicted by our findings.

Although the Onsager model is a very simple one and is based on rather strong assumptions, such as a spherical shape of the chromophore or a constant dipole moment with respect to the solvent, which are not fulfilled here, it usually succeeds in reproducing the order of magnitude of the phenomenon. It is also fair to remind that the definition of the Onsager radius relies on some arbitrary assumptions, in particular with respect to the definition of the boundaries used for its estimation.

As shown in Table 2 the calculated values of the ground state dipole moment range between 6.65 and 7.80 D depending on the polarity of the medium. On the other hand, a significant increase of the dipole moment is found in the luminescent, local state at its equilibrium geometry, with a maximum difference with those of the ground state amounting to 10 D. Therefore, we may confirm that the local excited state is, in this case, inducing a significant variation of the dipole moment magnitude as compared to the ground state. Interestingly, the increase of the polarity of the solvent further enhances the difference of the dipole moments. These counterintuitive phenomena, i.e., an important Stokes shift and the dipole moment increase in a purely local excited state, can be ascribed to the important geometrical relaxation of the locally excited state. This involves in particular an increase of the planarity of the conjugated systems with the dihedral between the phenyl and naphthalene moieties being increased to 176° for the most polar solvent and 173° for the apolar media (See also Supplementary Figure S1). Such a geometrical relaxation is again coherent with the fact that the locally excited state involves an extended conjugation pattern encompassing the two aromatic systems.

**TABLE 2:** Computed ground and excited state dipole moment in different environments.

	μ S_0_ (debye)	μ S_1_ (debye)	Δ μ (debye)
*in vacuo*	6.65	12.39	5.74
Hexane	7.16	14.35	7.19
THF	7.67	17.21	9.54
Dichloromethane	7.71	17.45	9.74
Ethanol	7.81	18.13	10.32
DMF	7.83	18.29	10.46
Acetonitrile	7.71	18.28	10.57

### 3.3 Emission properties in the presence of biological components

The extended aromatic system and marked hydrophobicity (low water solubility) of adapalene might favor its interaction with biomolecular assemblies such as proteins, DNA or membranes, and result in a shift of its emission band in relation with the polarity of the microenvironment probed by the fluorophore. Therefore, adapalene interaction with human serum albumin (HSA), salmon testes DNA, and cetyltrimethylammonium bromide micelles (CTAB), as a membrane mimic, was addressed by fluorescence spectroscopy.

**FIGURE 8:**
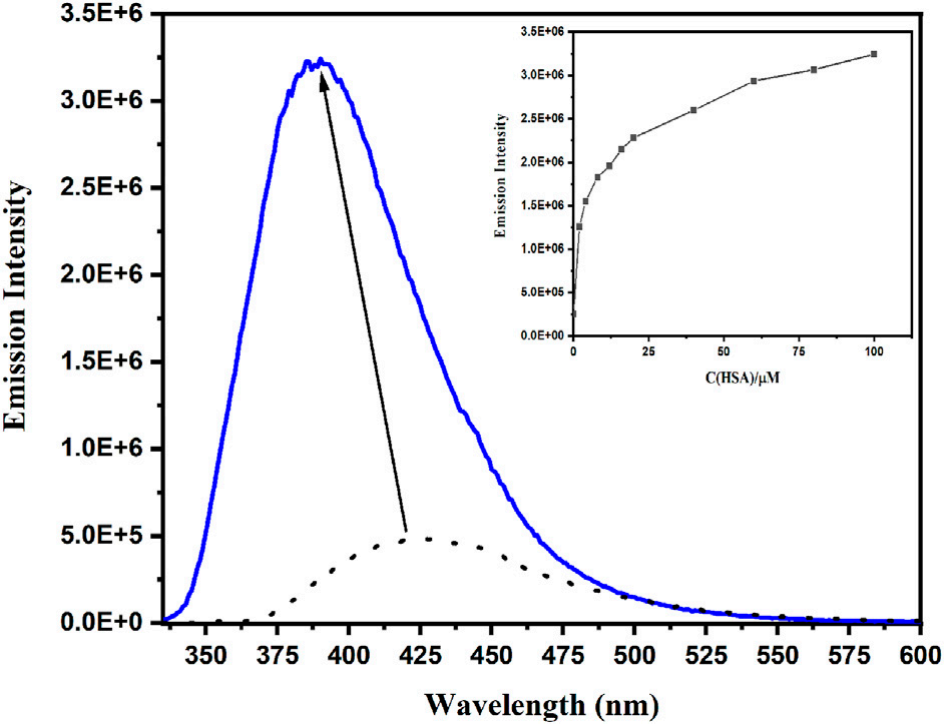
Fluorescence emission (λ_exc_ = 315 nm) of a solution containing adapalene (10 μM) and HSA (12 μM) in PBS at pH 7.4, adapalene alone in PBS (gray dotted line). Inset emission changes as a function of the protein concentration.

Human serum albumin (HSA) is a model of choice to study the binding properties of drugs, since this transport protein plays an essential role in the pharmacokinetics, pharmacodynamics, and toxicology of most of the xenobiotics (Merlot et al., 2014; Vayá et al., 2014). Thus, the binding of adapalene (10 μM) to HSA (from 2 to 100 μM) was studied by monitoring the changes in the emission spectra after selective excitation of the drug (λ_exc_ = 315 nm). Adapalene fluorescence in phosphate buffer (10 mM PBS, 10 mM NaCl, 0.1% DMSO) at pH 7.4 exhibits an emission maximum at 425 nm, which corresponds to its deprotonated form (pK_a_ ca. 4.2). As shown in Figure 8, a blue shifted and increased emission appears in the presence of HSA (λ_em_ = 390 nm). These emission changes are indicative of the encapsulation of adapalene to, at least, one of the protein hydrophobic cavities. As a matter of fact, the change of microenvironment probed by the fluorophore can be estimated from the standard plot of ET (30) vs. emission maxima (Figure 7). Encapsulated adapalene emits at λ_em_ of 390 nm, which corresponds to a ET (30) of ca. 36. This value is close to that previously described in the literature, ET (30) = 38.4, using an naphthalene-derived probe encapsulated in HSA (Singh et al., 2009). These results clearly indicate that the binding site is less polar compared to that of bulk PBS. In addition, the intensity increase might be related to the reduction of the nonradiative channels provided by protic solvents that induce a decrease of the fluorescence quantum yields through H-bonding interactions with the solvent molecules, even though some interaction with protein residues is probable.

The marked changes observed in the emission spectra can be used to quantitatively estimate the extent of binding to HSA by determining the binding constant, K, with Eq. 3, also known as the Benesi-Hildebrand equation (Benesi and Hildebrand, 1949; Singh et al., 2009). For this, a 1:1 complex is considered, the equilibrium can be expressed as:

$$\text{HSA}+\text{adapalene}\overset{K}{\underset{K^{-1}}{⇄}}\text{HSA}:\text{adapalene}$$

Where K can be written as K = [HSA:adapalene]/[HSA][adapalene]. Moreover, the assumption is made that the concentration of free HSA, [HSA], is much higher that the concentration of the 1:1 complex, [HSA:adapalene]. Then, the equation that applies is:

$$\frac{1}{\left(I-I_{0}\right)}=\frac{1}{\left(I_{i}-I_{0}\right)}+\frac{1}{\left(I_{i}-I_{0}\right)K\left[HSA\right]}$$ (3)

Where I_0_, I and I_i_ are the emission intensities in the absence of, at intermediate and infinite concentration of HSA. Therefore, the binding constant K can be obtained from the intercept and the slope of the linear fitting of the plot 1/(I-I_0_) vs. 1/[HSA] (Supplementary Figure S2). A value of K = 9.5 × 10^4^ M^−1^ is obtained.

**FIGURE 9:**
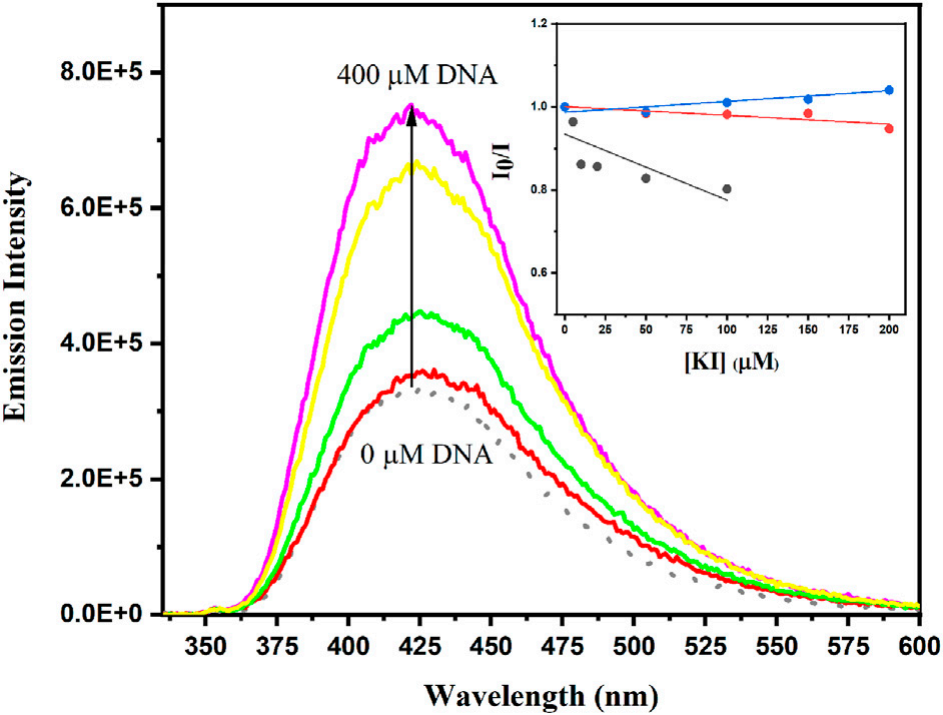
Fluorescence emission (λ_exc_ = 315 nm) of adapalene (10 μM) alone in PBS at pH 7.4 (black) and in the presence of duplex salmon sperm DNA (from 25 to 400 μM in bp). Inset: Stern-Volmer plot for the quenching of adapalene fluorescence in PBS (blue), with 10 μM bp (orange) or 100 μM bp (grey) of DNA.

DNA is another biomolecule of interest. Drug-DNA interaction is a significant factor in pharmacology as its double helix represents the target of many drugs currently in clinical use, especially for anticancer treatments (Sirajuddin et al., 2013). In this context, recent studies unveiled the potential of adapalene to trigger DNA damage and inhibit its repair that, among other processes, results in S-phase cell arrest in HaCat keratinocytes and melanoma cells (Li et al., 2019; Wang et al., 2020). This could be related to a high interaction of adapalene to the duplex with a reported binding constant of 1.01 × 10^5^ M^−1^ determined by UV-Vis spectrophotometry (Milanese et al., 2011). Here, the emission (λ_exc_ = 315 nm) of a mixture of adapalene (10 μM) and duplex salmon sperm DNA (from 25 to 400 μM in bp) was studied and compared to that of the drug in bulk PBS. As shown in Figure 9, addition of DNA results in an increased fluorescence intensity arising from drug interaction with the duplex. However, no significant shift of the emission maximum was evidenced. This results could be interpreted in two ways: the complexed drug i) does not experience an important change of its surroundings and is still exposed to bulk solution or ii) has an environment with a ET (30) ≈ 43 [obtained from the *ν*_F_ vs. ET (30) plot (Figure 7)]. The former hypothesis is incompatible with the intercalative binding of the phenyl-naphthyl system previously reported for adapalene (Milanese et al., 2011). As a matter of fact, the determined polarity index is similar to that given by Sinkeldam et al. for probes buried within a double helical B-form DNA, which points toward the encapsulation hypothesis (Sinkeldam et al., 2008). Indeed, spectrophotometric titrations of the interaction between adapalene derivatives and DNA concluded on the predominant role of the adamantyl group in the complexation process as the binding constant is decreased of one order of magnitude, K_a_ = 1.08 × 10^4^ M^−1^, in its absence. By contrast, esterification of the carboxylic acid function affects the binding, but, to a smaller extent (K_a_ = 7.4 × 10^4^ M^−1^) (Milanese et al., 2011). Thus, further experiments were run to conclude on the mode of interaction. Adapalene (10 μM) fluorescence quenching by iodide ion (Kumar and Asuncion, 1992; Khorasani-Motlagh et al., 2013) was monitored for the drug alone in PBS or in the presence of DNA (10 μM or 100 μM in bp). In bulk solution, a decrease of the emission is observed as expected for the enhancement of the intersystem crossing by the so-called heavy atom effect (Valeur and Berberan-Santos, 2012). A less efficient quenching occurs for the adapalene-DNA systems with a Stern-Volmer quenching constant 7.5 times lower for 1:1 mixture (Figure 9, inset). Consequently, adapalene is protected from iodide ion principally present in bulk solution, which is not compatible with electrostatic or groove interaction but with an intercalation (Milanese et al., 2011).

**FIGURE 10:**
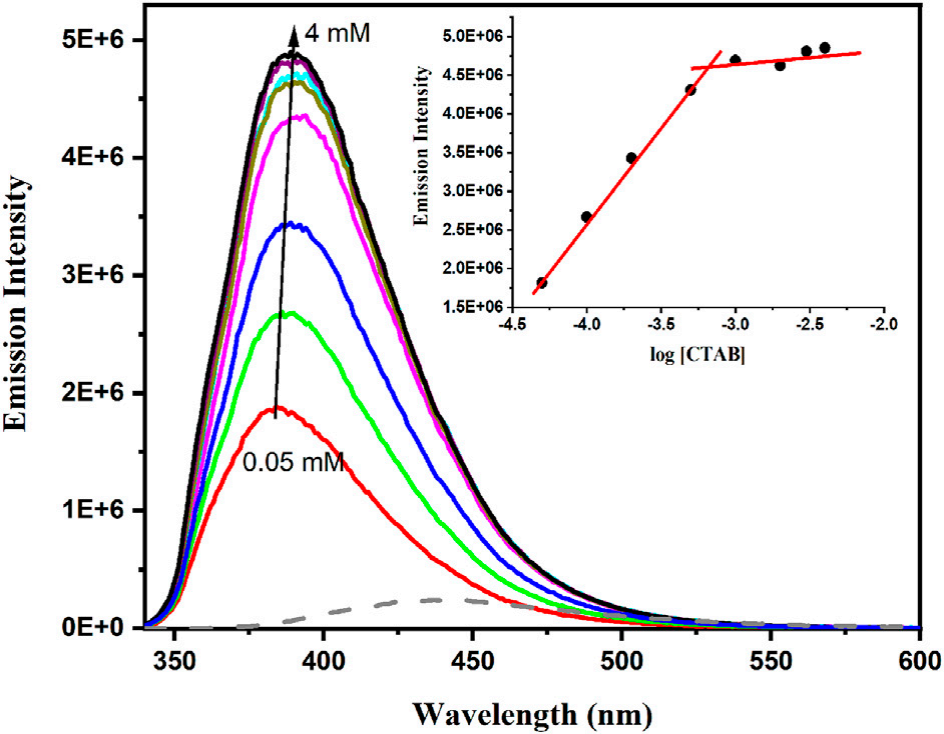
Emission spectra of adapalene (10 μM) varying CTAB concentration (from 0.05 to 4 mM) in PBS and adapalene alone in PBS (gray dotted line), λ_exc_ = 315 nm. Inset: Representation of the changes of 390 nm emission vs. the logarithm of CTAB concentration.

Finally, the interaction with membrane was addressed using CTAB micelles to mimic the native lipid bilayer environment. For this purpose, fluorescence spectra were registered (λ_exc_ = 315 nm) after the addition of increasing amounts of CTAB (from 0.05 to 4 mM) to an adapalene buffered solution at pH 7.4. A marked hypsochromic shift and hyperchromic effect appeared in the presence of CTAB (Figure 10). This behavior can be attributed to the incorporation of adapalene into the less polar environment of the micelles. In fact, using the data obtained from Figure 7A, the emission maximum corresponds to moderate polarity, with a polarity index ET (30) of ca. 35. In addition, representation of the change of fluorescence intensity *versus* the surfactant concentration shows a variation of the slope when micelles start to form (Figure 10, inset). Interestingly, the concentration corresponding to the intersection of the two slopes (ca. 0.5 mM) is in the same range that the reported CMC value for CTAB in PBS (Fuguet et al., 2005).

## 4 Conclusion

Absorption and emission properties of the widely used topical retinoid adapalene have been investigated paying a special attention to its solvent dependent behavior. This drug exhibits interesting features such as a UVB/UVA absorption band, together with a strong fluorescence emission and a marked positive solvatochromism. The usefulness of adapalene as a micro (bio) environment probe has been confirmed in the presence of albumin, DNA and CTAB. Molecular modeling and simulations reveal the presence of two quasi degenerated states corresponding to local and intermolecular charge-transfer states. Surprisingly, the emissive state is π-π* in nature. The counterintuitive results, i.e., important Stokes shift and solvatochromism associated to a local state, are ascribed to an important geometrical relaxation with an increase in the planarity of the system in the excited state. To summarize, selective UVB/UVA excitation of adapalene in the presence of biological components together with its polarity dependent emission makes it an interesting probe to investigate its location in biological media.

## Data Availability

The original contributions presented in the study are included in the article/Supplementary Material, further inquiries can be directed to the corresponding author.
