# Supplementary material for: Environmentally sensitive fluorescence of the topical retinoid adapalene
Source: Front Chem. 2024 Jul 8;12:1438751. doi: 10.3389/fchem.2024.1438751 (PMC11260622; doi:10.3389/fchem.2024.1438751)
Supplement: Supplementary file 1 [file DataSheet1.PDF]

Supplementary Material

Index :

Table S1. Calculated Vertical Transitions for Absorption and Emission in Different Solvents. The Stoke Shifts is calculated as the difference between the emission vertical transitions and the average of the two lowest vertical excitation energies for absorption. Oscillator strengths are given in parenthesis

Figure S1. Optimized geometries for the ground (A) and the lowest excited state B) showing the increased planarity

Figure S2. Benesi-Hildebrand plot obtained from fluorescence emission (λ_{exc}= 315 nm) of a solution containing adapalene (10 µM) and HSA (from 2 to 100 µM) in PBS at pH 7.4

Table S1. Calculated Vertical Transitions for Absorption and Emission in Different Solvents. The Stoke Shifts is calculated as the difference between the emission vertical transitions and the average of the two lowest vertical excitation energies for absorption. Oscillator strengths are given in parenthesis.

	Absorption (nm)	Emission (nm)	Stoke Shift (10^3 cm^{-1})
in vacuo	275.3 (0.0)<br>270.4 (0.5)	318.2 (0.9)	5.23
Hexane	277.1 (0.3)<br>275.5 (0.3)	342.4 (1.3)	6.99
THF	280.3 (0.5)<br>276.7 (0.2)	366.7 (1.5)	8.64
Dichloromethane	280.7 (0.5)<br>276.8 (0.2)	368.9 (1.4)	8.77
Ethanol	280.9 (0.3)<br>276.9 (0.3)	374.6 (1.6)	9.16
DMF	281.6 (0.6)<br>277.1 (0.2)	376.0 (1.6)	9.20
Acetonitrile	280.9 (0.5)<br>276.9 (0.2)	375.8 (1.6)	9.25

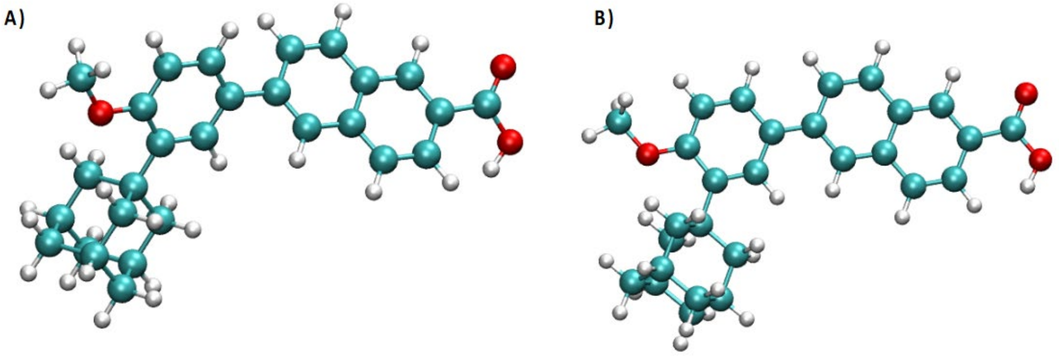

Figure S1. Optimized geometries for the ground A) and the lowest excited state B) showing the increased planarity.

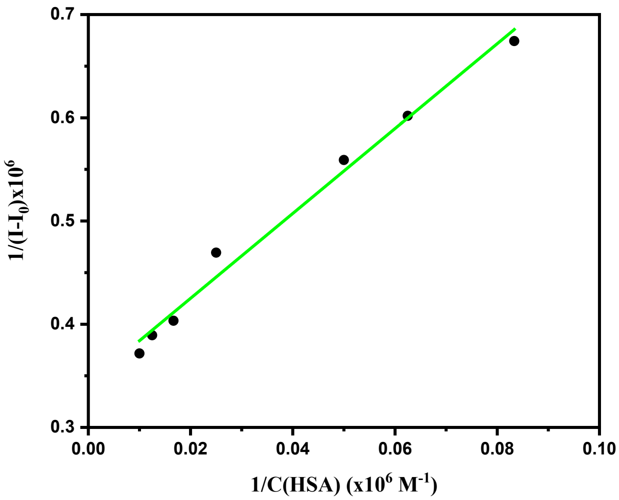

Figure S2. Benesi-Hildebrand plot obtained from fluorescence emission (λ_{exc}= 315 nm) of a solution containing adapalene (10 μM) and HSA (from 2 to 100 μM) in PBS at pH 7.4.
